# A single-step protocol for closing experimental atom balances

**DOI:** 10.1016/j.mex.2020.100781

**Published:** 2020-01-10

**Authors:** Philippe M. Heynderickx

**Affiliations:** a Center for Environmental and Energy Research (CEER) – Engineering of Materials Via Catalysis and Characterization, Ghent University Global Campus, 119-5 Songdomunhwa-Ro, Yeonsu-Gu, Incheon, 406-840, South Korea; b Department of Green Chemistry and Technology, Faculty of Bioscience Engineering, Ghent University, 653 Coupure Links, Ghent, B-9000, Belgium

**Keywords:** CLOBAL – after ‘closing the atom balances’, which is exactly what the presented procedure does, Closing atom balances, Accuracy, Excel® data sheet

## Abstract

Molar balances are considered to be closed if they are within 95–105%. It was shown in the companion paper “https://doi.org/10.1016/j.cej.2018.12.113; Chem. Eng. J., 361, 805–811 (2019)” that even this condition can give rise to pronounced deviations in conversion or selectivity data (Heynderickx, 2019). This manuscript offers a very simple a posteriori calculation procedure to address these deviations via simple linear algebra. The specific details of this procedure, called ‘CLOBAL’, after ‘closing the balances’, are shared (1) by showing the mathematics behind-the-scene and (2) by showing the specific programming code with an itemized guideline through the code.

Key benefits of proposed procedure CLOBAL script are:

• Physical quantities such as molar flow rates, concentrations or absolute number of moles are updated via a one-step linear procedure to close the corresponding atom balances;
• The presented CLOBAL procedure, is executed in Excel®, which is accessible and practical for every user – no need for special license and the code is provided; and
• Parameter estimation, using treated data, results in smaller confidence intervals and lower residual sum of squares (RSSQ).

## Graphical abstract

**Figure:**
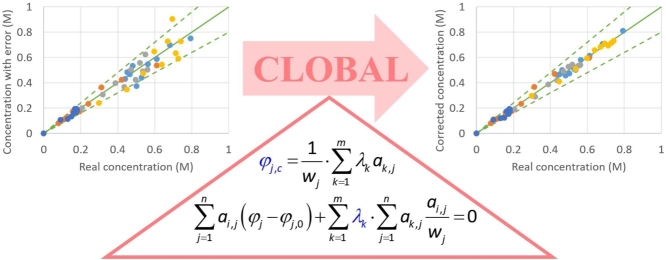

**Specification Table**

**Table:** 

Subject Area:	Chemical Engineering
More specific subject area:	Fields with experimental outcomes such as molar flow rates, concentrations, moles in organic chemistry experiments, catalysis…
Method name:	CLOBAL – after ‘closing the atom balances’, which is exactly what the presented procedure does
Name and reference of original method:	P. M. Heynderickx, Closing the balance by the CLOBAL procedure: towards more accurate concentration, conversion and selectivity values, https://doi.org/10.1016/j.cej.2018.12.113
Resource availability:	Example of customized procedure is given in file clobal_01.xlsm

## Method details

When chemical reactions are performed the corresponding element or atom balances should be always closed [[1], [2], [3], [4], [5]]. For example, if the carbon balance is envisaged in a non-nuclear reaction, the initial number of carbon moles should equal the carbon in the reaction products. Typical acceptable ranges for an atom balance are between 90 % and 110 %. Experimental error is logically invoked to explain why atom balances are not exactly equal to 100 %.

This manuscript describes a very simple and elegant method to set atom balances equal to 100 %. Striking consequence of the given CLOBAL procedure is a more accurate calculation of conversion and selectivity values and a lower residual sum of squares during parameter estimation, accompanied by smaller confidence intervals for the parameters [1].

Consider n measurements of n physical quantities, which ‘true’ values are called φ_j_, j = 1…n. For the sake of example, these quantities are the outlet molar flow rates in a mixture of n compounds, A_j_. Each of these compounds A_j_ has a_i,j_ atoms of type e_i_, i = 1… m. Normally the number of compounds exceeds the number of elements taken into account, i.e., m < n. Since there are no nuclear reactions or transformations included, Eq. (1) holds for the true values with φ_j,0_ the initial value for quantity φ_j_:

(1) $$\begin{array}{cc} \sum_{j=1}^{n}\;a_{i,j}\varphi_{j,0}=\sum_{j=1}^{n}\;a_{i,j}\varphi_{j} & \text{i}=1\ldots \text{m} \end{array}$$

Eq. (1) is an ideal representation, i.e., all the balances for atom type e_i_, i = 1… m, are 100 % closed.

In reality this is not the case due to experimental error and, hence, the experimental values for the molar flow rate, absolute number of moles or concentrations do not close Eq. (1). The purpose of this manuscript is to offer a method for small corrections on these physical quantities in order to close the balances 100 %. The order of magnitude of these corrections can be compared to the error related to typical calibration data, as outlined in the companion paper [1], and, if the calibration curve has a high R^2^, subsequently small corrections to the concentrations, mol fractions, or derived flowrates, are to be expected with this method. The proposed correction on the physical quantity, φ_j,c_ with j = 1…n, should result in a full closure of the m balances, so that Eq. (2) is valid:

(2) $$\begin{array}{cc} \sum_{j=1}^{n}\;a_{i,j}\varphi_{j,0}=\sum_{j=1}^{n}\;a_{i,j}\left(\varphi_{j}+\varphi_{j,c}\right) & \text{i}=1\ldots \text{m} \end{array}$$

Eq. (2) represents m so-called ‘fundamental relations’ for the n corrections φ_j,c_. Hence, n–m additional relations are required to solve for all of their values. These can be found from Eq. (3), which states that the weighted sum of corrections should be minimal, with w_j_ the weight factor corresponding for correction φ_j,c_:

(3) $$R=\sum_{j=1}^{n}\;w_{j}\varphi_{j,c}^{2}\;\to \;min$$

Eqs. (2) and (3) form the basis for a so-called ‘Lagrange multiplicator optimization problem’: R needs to be minimized and the solution is subjected to equality constraints, see Eq. (2). The great advantage of the Lagrange multiplicator method is that it allows not to explicitly solve the constraint equations and use them to eliminate extra variables. The complete function, also called the Lagrangian function S [6], with the so-called ‘Lagrange multiplicators’, 2·λ_i_ (i = 1…m), which has to be minimized, reads as Eq. (4):

(4) $$S=\sum_{j=1}^{n}\;w_{j}\varphi_{j,c}^{2}+\sum_{i=1}^{m}\;2\lambda_{i}\left(\sum_{j=1}^{n}\;a_{i,j}\varphi_{j,0}-\sum_{j=1}^{n}\;a_{i,j}\left(\varphi_{j}+\varphi_{j,c}\right)\right)\;\to \;\min$$

The prefactor ‘2’ for the equality constraint can be added for the sake of elegancy, so that in further calculations the factor 2, as a result of the derivative of the quadratic function (3), can be cancelled out.

Taking the derivative with respect to φ_j,c_, gives Eq. (5):

(5) $$\begin{array}{cc} \frac{\partial S}{\partial \varphi_{j,c}}=2\;w_{j}\varphi_{j,c}-\sum_{i=1}^{m}\;2\lambda_{i}\;a_{i,j}=0 & \text{j}=1\ldots \text{n} \end{array}$$

From Eq. (5) the optimized corrections for the n flow rates, φ_j,c_, are given by Eq. (6):

(6) $$\begin{array}{cc} w_{j}\varphi_{j,c}=\sum_{k=1}^{m}\;\lambda_{k}\;a_{k,j} & \text{j}=1\ldots \text{n} \end{array}$$

Eq. (6) contains n relations and m + n unknowns, hence, m additional relations are needed, which can be found in Eq. (2). The subsequent substitution of Eq. (6) in the latter gives Eq. (7):

(7) $$\begin{array}{cc} \sum_{j=1}^{n}\;a_{i,j}\left(\varphi_{j}-\varphi_{j,0}\right)+\sum_{k=1}^{m}\;\lambda_{k}\cdot \sum_{j=1}^{n}\;a_{k,j}\frac{a_{i,j}}{w_{j}}=0 & \text{i}=1\ldots \text{m} \end{array}$$

Eq. (7) represents a set of m linear relations for λ_k_, i = 1…m, is found and upon solving, the Lagrange multiplicators are inserted into Eq. (6) to obtain the individual correction for each of the individual n molar flow rates:

(8) $$\begin{array}{cc} \varphi_{j,c}=\frac{1}{w_{j}}\cdot \sum_{k=1}^{m}\;\lambda_{k}\;a_{k,j} & \text{j}=1\ldots \text{n} \end{array}$$

The corrected quantities φ_j_ + φ_j,c_, for j = 1…n, give complete balances (1). Expressions (7) and (8) are sufficiently detailed to replicate the presented CLOBAL protocol.

The given expressions (7) and (8) can be written in general matrix notation, which will form the basis of the Excel® macro that gives the corrections.

In order to validate the presented methodology, the condensation of benzaldehyde and heptanal, which is an important aldol-type reaction in the production of jasmine aldehyde [[7], [8], [9]], is taken as showcase in the companion paper [1]. There are 5 compounds to be considered: benzaldehyde (C_7_H_6_O), heptanal (C_7_H_14_O), jasmine aldehyde (C_7_H_14_O), as desired product, and water (H_2_O) and the dimer 2-pentyl-2-nonenal (C_14_H_26_O) as by-product (n = 5). Three atom types are used: C, O and H (m = 3), so that the stoichiometric matrix, allocating all coefficients a_i,j_, is given by Eq. (9):

(9) $$\underline{\underline{a}}=\left(\begin{array}{ccc} 7 & 1 & 6\\ 7 & 1 & 14\\ 14 & 1 & 18\\ 0 & 1 & 2\\ 14 & 1 & 26 \end{array}\right)$$

The difference in actual value and initial value is given by vector $\underline{\Phi }$, see Eq. (10), and the correction vector is defined by Eq. (11):

(10) $$\begin{array}{cc} {\left(\underline{\Phi }\right)}_{j}=\varphi_{j,0}-\varphi_{j} & \text{j}=1\ldots \text{n} \end{array}$$

(11) $$\begin{array}{cc} {\left(\underline{\tilde{\Phi }}\right)}_{j}=\varphi_{j,c} & \text{j}=1\ldots \text{n} \end{array}$$

The solution for the m Lagrange multiplicators is given by Eq. (12) with substitution of matrix $\underline{\underline{v}}$, see Eq. (13):

(12) $$\underline{\lambda }={\left({\underline{\underline{a}}}^{T}\underline{\underline{v}}\right)}^{-1}{\underline{\underline{a}}}^{T}\underline{\Phi }$$

(13) $$\begin{array}{cc} {\left(\underline{\underline{v}}\right)}_{i,j}=\frac{1}{w_{j}}\cdot {\left(\underline{\underline{a}}\right)}_{i,j} & \text{i}=1\ldots \text{n},\text{ j}=1\ldots \text{m} \end{array}$$

Eq. (12) represents the solution of Eq. (7) in matrix notation with respect to the Lagrange multiplicators.

The corrections φ_j,c_ for j = 1…n are given by Eq. (14) in one single step calculation, i.e., no iterations are required:

(14) $$\underline{\tilde{\Phi }}=\underline{\underline{v}}\;\underline{\lambda }=\underline{\underline{v}}{\left({\underline{\underline{a}}}^{T}\underline{\underline{v}}\right)}^{-1}{\underline{\underline{a}}}^{T}\underline{\Phi }$$

**Fig. 1:**
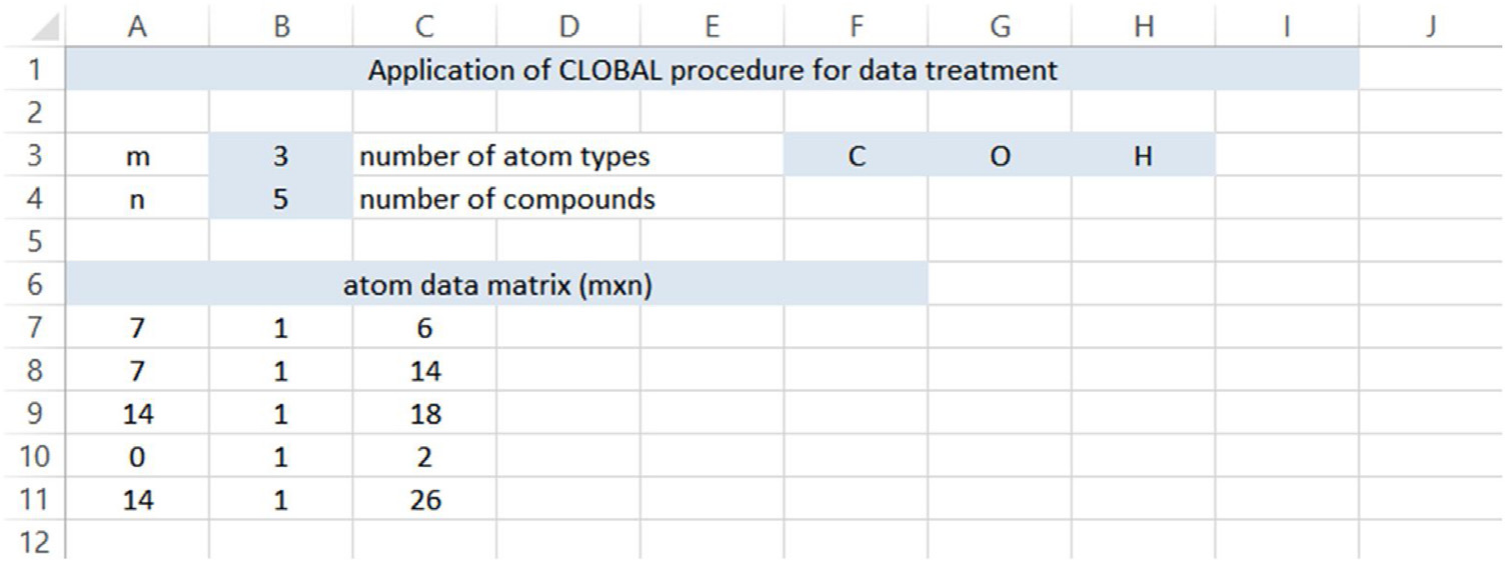
Input sheet ‘atom’ for CLOBAL procedure: information on atom types and input of stoichiometry.

The corresponding VBA code is given in Table 1. The input requires the number of atom types, m, and the number of compounds, n. The stoichiometric information on the atom types in the individual compounds, such as given by the stoichiometric matrix via Eq. (9), is the input in worksheet ‘atom’, see Fig. 1. On the third row, the elements are given for further use in the results sheet. In this case the carbon, oxygen and hydrogen balance are evaluated (C, O and H). The code is divided in sections:

• Row 1 to 2: start of the routine;
• Row 3 to 14: declaration of variables;
• Row 15 to 16: removing previous results (avoiding erroneous overlap in data treatment);
• Row 17 to 28: reading input from ‘atom’ sheet;
• Row 29 to 34: reading input from ‘data’ sheet;
• Row 35 to 48: textual setting in the ‘result’ sheet in order to receive the results;
• Row 49 to 55: CLOBAL procedure starts by transposing the stoichiometric matrix (9);
• Row 56 to 76: all inputted data are treated (ii = 1…ndata) according to Eqs. (10), (11), (12), (13), (14):
  ○ x1 contains the elements of vector $\underline{\Phi }$, see Eq. (10);
  ○ x2 contains the elements for matrix $\underline{\underline{v}}$, see Eq. (13);
  ○ x3 is the transposed of matrix $\underline{\underline{a}}$;
  ○ x4 represents ${\underline{\underline{a}}}^{T}\underline{\underline{v}}$;
  ○ x5 represents ${\underline{\underline{a}}}^{T}\underline{\Phi }$;
  ○ x6 contains the Lagrange multiplicators, calculated via Eq. (12); and
  ○ x7 contains the correction on the given physical quantities (in this case, concentrations), calculated via Eq. (14);
• Row 77 to 97: allocation of all the results;
• Row 98: end of the loop over all ndata; and
• Row 99: End of the routine

**Table 1:** Excel® code for the CLOBAL procedure.

**Fig. 2:**
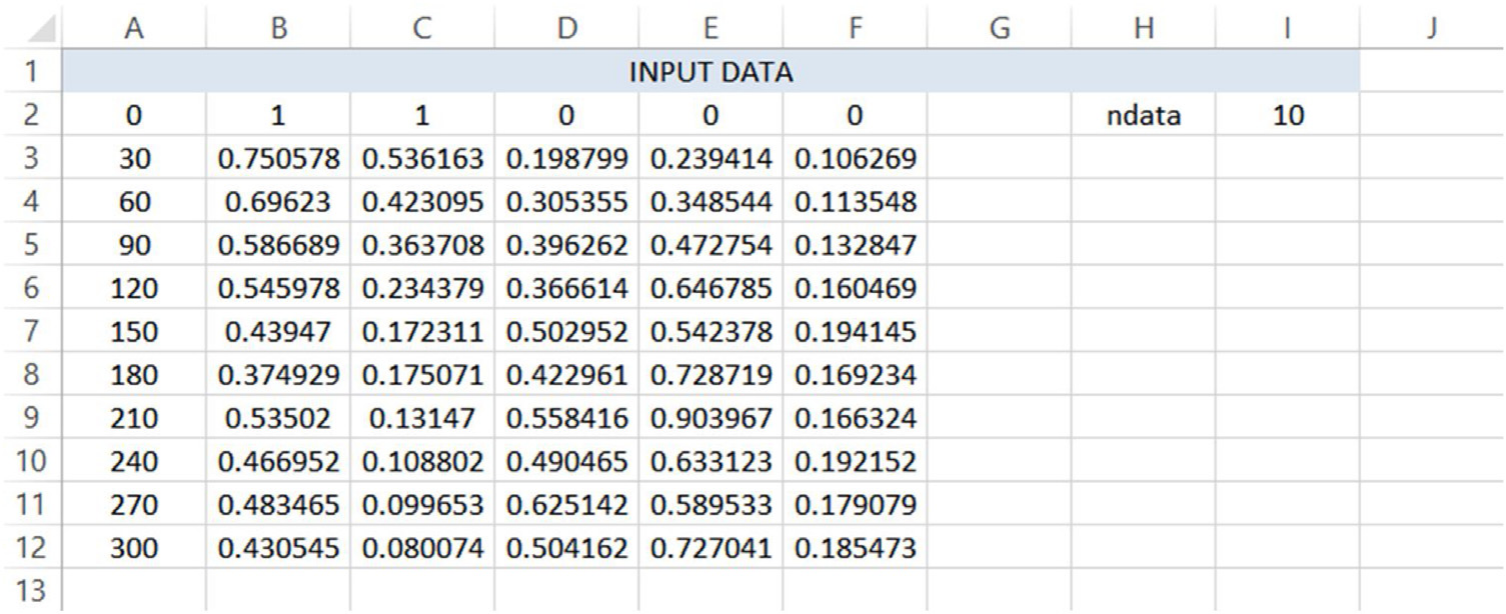
Input sheet ‘data’ for CLOBAL procedure: experimental data, corresponding to initial conditions in the companion paper [1] (CB,0 = 1 M, CH,0 = 2 M), see Fig. 5.

The data vector consists of ndata+1 rows, having the initial concentration on row 2, see Fig. 2. The value of ‘ndata’ is automatically read by the program, depending on the input in the worksheet ‘data’; maximal number of data is n_max, n_max = 1000. The actual concentration values for the n compounds occupy the rows 3 to ndata+2. The first column in worksheet ‘data’ contains the independent variable, e.g., in this case the minutes at sampling. This can be used for preparation of figures, but for the given procedure it is not required.

**Fig. 3:**
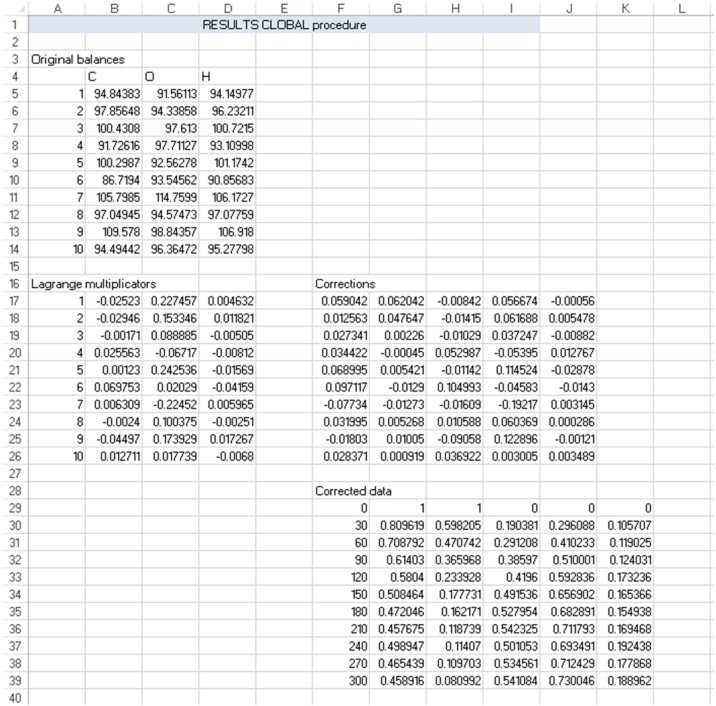
Results sheet ‘results’ for CLOBAL procedure, corresponding to initial conditions in the companion paper [1] (CB,0 = 1 M, CH,0 = 2 M), see Fig. 5.

Fig. 3 gives the results of the CLOBAL procedure: worksheet ‘results’ evaluates the original atom balances and feeds this back to the user on rows 3 to ndata+4. The Lagrange multiplicators, calculated via Eq. (12), and the individual corrections, obtained via Eq. (14), are given on rows ndata+6 to 2*ndata+6. The corrected data are given from row 2*ndata+8 to 3*ndata+9 and they are ready for further use, i.e., they are generated as in the input form for sheet ‘data’.

As a side note for the weight factors, the author found that the best choice is the inverse of the corresponding response; as indicated on line 60 of the code, see Table 1. This can be altered by the user in case another expression should be more appropriate.

**Fig. 4:**
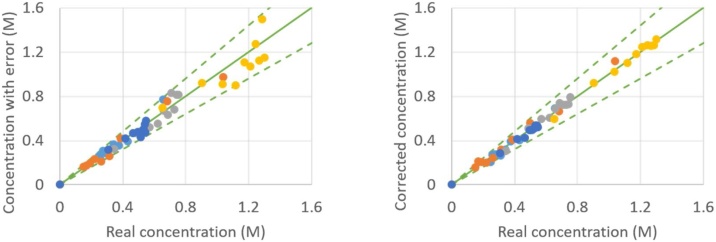
Concentration with average 10 % error (left) and concentration after CLOBAL procedure (right) versus real concentration. () B (benzaldehyde), () H (heptanal), () J (jasmin aldehyde), () W (water), () D (2-pentylhept-2-enal) with CB,0 = 1.0 M, CH,0 = 2.0 M, others = 0.0 M [1]. Full green line is the first bisector; dashed lines represent ±20 error.

**Fig. 5:**
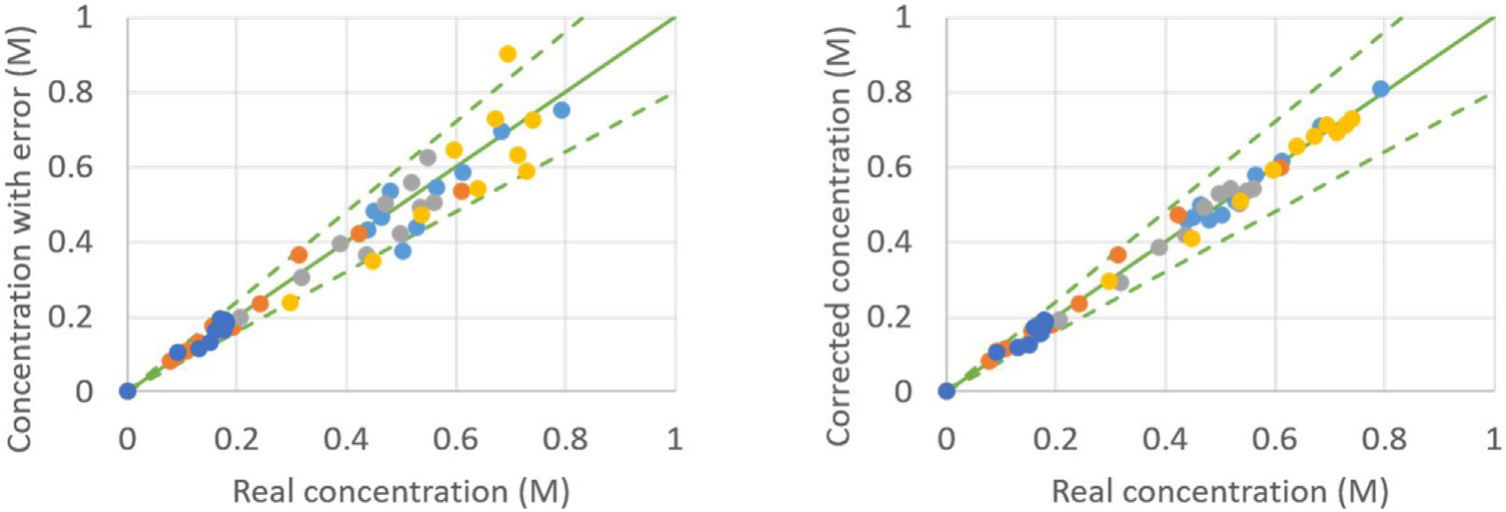
Concentration with average 10 % error (left) and concentration after CLOBAL procedure (right) versus real concentration. () B (benzaldehyde), () H (heptanal), () J (jasmin aldehyde), () W (water), () D (2-pentylhept-2-enal) with CB,0 = 1.0 M, CH,0 = 1.0 M, others = 0.0 M [1]. Full green line is the first bisector; dashed lines represent ±20 error.

**Fig. 6:**
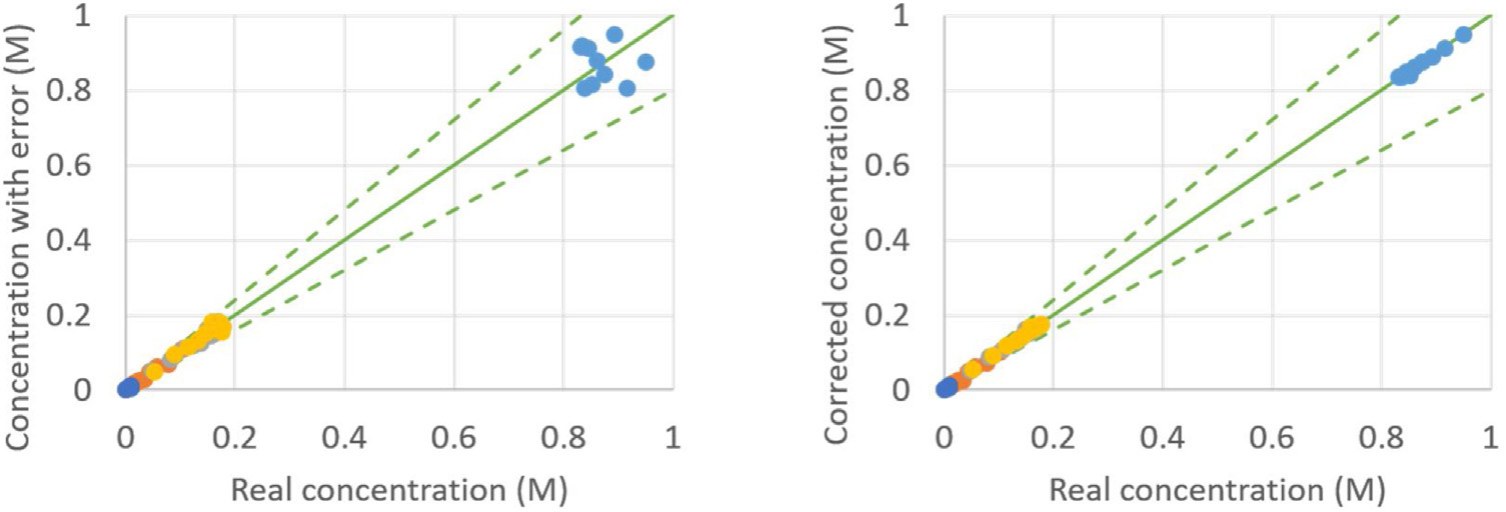
Concentration with average 10 % error (left) and concentration after CLOBAL procedure (right) versus real concentration. () B (benzaldehyde), () H (heptanal), () J (jasmin aldehyde), () W (water), () D (2-pentylhept-2-enal) with CB,0 = 1.0 M, CH,0 = 0.2 M, others = 0.0 M [1]. Full green line is the first bisector; dashed lines represent ±20 error.

**Fig. 7:**
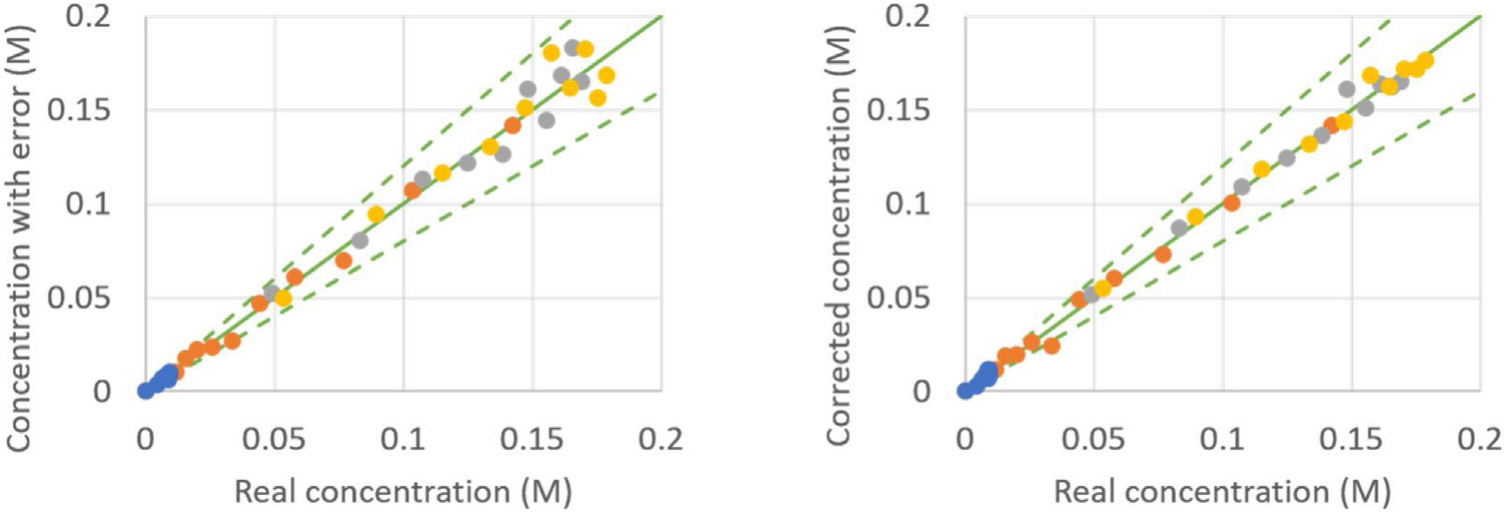
Concentration with average 10 % error (top) and concentration after CLOBAL procedure (bottom) versus real concentration: zoom of Fig. 6 for concentration range 0 to 0.20 M.

As an example, the result of the proposed procedure is given in Fig. 4, Fig. 5, Fig. 6, Fig. 7, from which a clear overall decrease in data spread is observable. It has to be mentioned that some points might not show any improvement, such as the point (0.30 M; 0.35 M) in Fig. 5 or the point (0.035 M; 0.024 M) in Fig. 7. This is purely a coincidence: when the in silico random error is applied a second time [10] and the CLOBAL procedure is subsequently applied, the balances are still closed, but the small variations are somewhat different due to the different randomized error; this time resulting in a visible improvement of the point of interest. It was shown in the companion paper [1] that parameter estimation via ODRpack [11], using treated data, results in smaller confidence intervals and lower residual sum of squares (RSSQ).

## Declaration of Competing Interest

The author declares that they have no known competing financial interests or personal relationships that could have appeared to influence the work reported in this paper.

## References

[1] Heynderickx P.M. (2019). Closing the balance by the CLOBAL procedure: towards more accurate concentration, conversion and selectivity values. Chem. Eng. J..

[2] Froment G.F., Bischoff K.B. (1990). Chemical Reactor Analysis.

[3] Heynderickx P.M. (2018). Acquisition of nonlinear kinetics from linear relations: application on homogeneous transesterification reactions. Chem. Eng. J..

[4] Heynderickx P.M., Thybaut J.W., Poelman H., Poelman D., Marin G.B. (2010). Kinetic modeling of the total oxidation of propane over CuO-CeO_2_/g-Al_2_O_3_. Appl. Catal. B: Environ..

[5] Heynderickx P.M., Thybaut J.W., Poelman H., Poelman D., Marin G.B. (2009). Kinetic modeling of the total oxidation of propane over anatase and vanadia sputter deposited catalysts. Appl. Catal. B: Environ..

[6] Luenberger D.G. (1969). Optimization by Vector Space Methods.

[7] Sharma S.K., Parikh P.A., Jasra R.V. (2008). Eco-friendly synthesis of jasminaldehyde by condensation of 1-heptanal with benzaldehyde using hydrotalcite as a solid base catalyst. J. Mol. Catal. A.

[8] Sharma S.K., Parikh P.A., Jasra R.V. (2010). Reconstructed Mg/Al hydrotalcite as a solid base catalyst for synthesis of jasminaldehyde. Appl. Catal. A: Gen..

[9] Sudheesh N., Sharma S.K., Khokhar M.D., Shukla R.S. (2011). Kinetic investigations on the modified chitosan catalyzed solvent-free synthesis of jasminaldehyde. J. Mol. Catal. A.

[10] Heynderickx P.M., Roelant R. (2018). Superposition of artificial experimental error onto calculated time series: construction of in-silico data sets. Data Brief.

[11] Boggs P.T., Byrd R.H., Schnabel R.B. (1987). A stable and efficient algorithm for nonlinear orthogonal distance regression. SIAM J. Sci. Stat. Comput..

